# Parasitic Developments in the Human Body
*On Cystic Entozoa in the Human Kidney. By T. Herbert Barker, M.D. London: 1856. Hamilton and Adams.


**Published:** 1856-04

**Authors:** 


### THE EPITOME OF SANITARY LITERATURE.

### PARASITIC DEVELOPMENTS IN THE HUMAN BODY.*

WITHIN these last two years, great advances have been made in relation to the development of parasites in the bodies of men and animals. It has long been known that the class of animals known as the entozoa are by no means uncommon occupants of the animal body. They exist in several conditions; among which are the cestoids, illustrated by the common tape-worm; and the cystic growths in which the entozoa are only partially developed. Dr. BARKER, in his interesting paper, has related a case, in which the cystic entozoa were developed in the kidney of a patient under his care.

A point of great interest to the sanitarian, on which Dr. Barker dwells, is, that the cystic entozoa may be taken into the body in food, and that thus received they may and do give rise to tape-worm; and *vice versâ*, that the introduction into the body of the segments or the ova of tape-worms, may if thus taken give origin to the cystic variety. The organs of the body, in which the cystic entozoa are most commonly located, are the liver, the cellular tissue, the muscles, the chambers of the eye, the brain, and the abdominal cavity. In the human subject, fatal results rarely occur from the presence of cystic entozoa; but Schleusner, in his *Medical Topography of Iceland*, describes that the inhabitants of that island are at this time suffering from the presence of these entozoa in a remarkable degree. The liver, peritoneum, and subcutaneous tissues, are the parts of the body most commonly attacked. About a sixth of the whole population are thus affected; and the result is, a long protracted illness, terminating in death. Von Siebold, a distinguished authority, thinks that the disease thus occurring is occasioned by the people swallowing accidentally the ova of tape-worms thrown off by dogs, of which immense numbers are kept in the island.

Dr. Barker's little work is a most useful addition to sanitary literature.

### NOTES ON THE MINING DISTRICTS.†

FROM a Report (1855) by Mr. H. S. TREMENHEERE, the

---

* On Cystic Entozoa in the Human Kidney. By T. HERBERT BARKER, M.D. London: 1856. Hamilton and Adams.

